# High-fat diet disrupts metabolism in two generations of rats in a parent-of-origin specific manner

**DOI:** 10.1038/srep31857

**Published:** 2016-08-23

**Authors:** T. J. G. Chambers, M. D. Morgan, A. H. Heger, R. M. Sharpe, A. J. Drake

**Affiliations:** 1 MRC Centre for Reproductive Health, University of Edinburgh, The Queen’s Medical Research Institute, 47 Little France Crescent, EH16 4TJ, UK; 2 MRC Computational Genomics Analysis and Training, Department of Physiology, Anatomy and Genetics, University of Oxford, Parks Road, Oxford, OX1 3PT, UK; 3 University/BHF Centre for Cardiovascular Science, University of Edinburgh, QMRI, 47 Little France Crescent, EH16 4TJ, UK

## Abstract

Experimental and epidemiological evidence demonstrate that ancestral diet might contribute towards offspring health. This suggests that nutrition may be able to modify genetic or epigenetic information carried by germ cells (GCs). To examine if a parental high fat diet (HFD) influences metabolic health in two generations of offspring, GC-eGFP Sprague Dawley rats were weaned onto HFD (45% fat) or Control Diet (CD; 10% fat). At 19 weeks, founders (F0) were bred with controls, establishing the F1 generation. HFD resulted in 9.7% and 14.7% increased weight gain in male and female F0 respectively. F1 offspring of HFD mothers and F1 daughters of HFD-fed fathers had increased weight gain compared to controls. F1 rats were bred with controls at 19 weeks to generate F2 offspring. F2 male offspring derived from HFD-fed maternal grandfathers exhibited increased adiposity, plasma leptin and luteinising hormone to testosterone ratio. Despite transmission via the founding male germline, we did not find significant changes in the F0 intra-testicular GC transcriptome. Thus, HFD consumption by maternal grandfathers results in a disrupted metabolic and reproductive hormone phenotype in grandsons in the absence of detectable changes in the intra-testicular GC transcriptome.

The prevalence of obesity has doubled since 1980 with an estimated $28billion annual increase in associated medical costs in the USA^1^,^2^,^3^. Whilst lifestyle influences obesity risk, other factors can contribute to weight accumulation and its effects on general health. Twin studies suggest that 40–70% of bodyweight can be explained by inherited factors^4^, however, with a few exceptions, specific genes have remained elusive and genome wide association studies have only accounted for 2–4% of the heritability of obesity.

Several human studies have identified relationships between parental weight and weight of offspring^5^, albeit this is confounded by parental and offspring environmental exposures. Experimental evidence suggests that this might occur via non-genetic mechanisms. For example, the diet of a male prior to conception can impact upon the metabolic and reproductive health of his offspring^6^,^7^,^8^,^9^, with some studies showing that such ‘programmed’ effects are transmissible to further generations^10^,^11^,^12^. It has been proposed that such environmental exposures might affect the germline epigenome; for example the miRNA profile^6^,^13^,^14^, chromatin dynamics/histone modifications^8^,^9^ and DNA methylome^12^ of spermatozoa. However, the mechanisms linking these germline epigenetic modifications, the stage of spermatogenesis affected and the phenotypic changes observed in offspring are unknown.

The present study shows that grandparental exposure to a high-fat diet influences the metabolism of two generations of rats in a grandparent-of-origin and sex-specific manner. We demonstrate that the maternal grand-sire has the strongest effect on the metabolic phenotype of his male grand-offspring. Using a rat model in which germ cells (GCs) express eGFP^15^, enabling the isolation of a pure intra-testicular germ cell population, we did not identify any changes to the gene-coding or miRNA transcriptome of GCs from the testes of HFD exposed adult males, implying that other (e.g. downstream) HFD-induced changes must account for the intergenerational effects which we identify.

## Results

### HFD induces adiposity but not altered glucose tolerance in exposed animals (F0)

**Figure 1:**
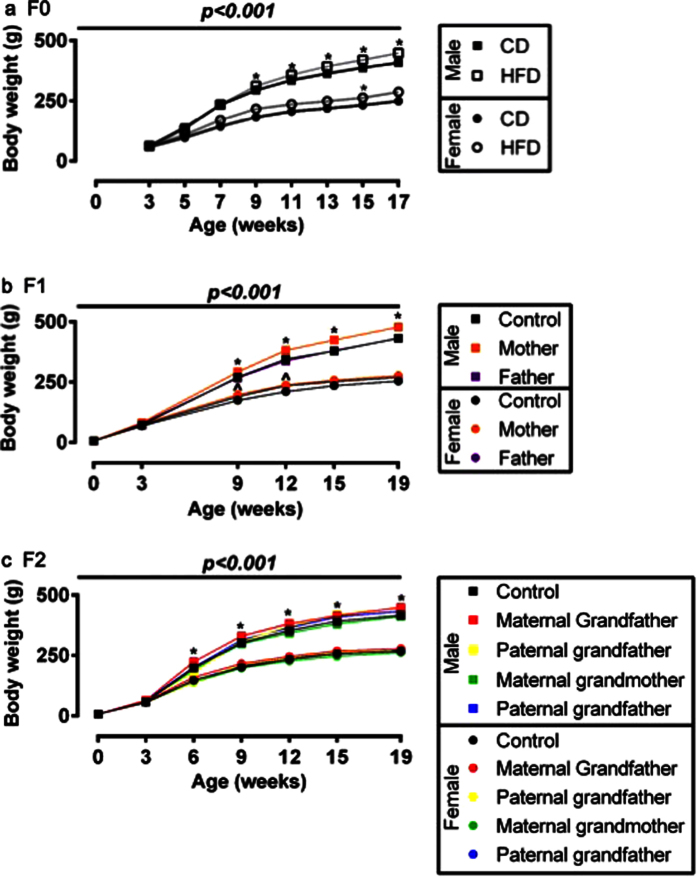
Growth curves of rats in each generation. (**a**) Body weight of F0 rats fed a control diet (CD; black lines, closed symbols) or a high fat (HFD; grey lines, open symbols) for 14 weeks. Data are shown for male (squares) and female (circles) rats. Data were analysed by linear mixed model with diet and sex as fixed factors, time as a repeated factor and cohort as a random factor with Bonferroni post hoc testing for effect of diet within a sex. N = 22–35 males and 10–18 females from two cohorts. *p < 0.05 HFD vs CD. (**b**) Body weight of F1 rats according to diet of the F0 parents. Analysis was by linear mixed model with group and sex as fixed factors and litter as a random factor. N = 13–32 males from 5–9 litters and 10–21 females from 5–9 litters. *p < 0.05 maternal high fat diet vs. both parents on control diet. ^p < 0.05 maternal or paternal high fat diet vs. both parent control diet. (**c**) Bodyweight of F2 rats from birth to 18 weeks of age. Analysis was by linear mixed model with sex and group as fixed factors, time as a repeated factor and litter as a random factor and post hoc Bonferroni analysis. *p < 0.05 maternal grandfather high fat diet vs. control. Data are means ± SEM.

From weaning male and female rats were placed onto a control diet (CD) or onto a high fat diet (HFD) for 16 weeks. Following 14 weeks on a HFD, F0 males and females were respectively 9.3% and 14.7% heavier compared to animals fed the CD, with a significant difference in male weight from 9 weeks of age and in female weight from 15 weeks (Fig. 1A). In males, the adiposity index (the sum of fat pads divided by body weight) was increased by 36% and leptin increased 3-fold, although this was not statistically significant (Table 1). Insulin secretion in response to glucose tolerance testing (GTT) was increased 150% in HFD fed males, without any changes in glucose concentration. There were no differences between groups in plasma lipids or triglycerides, or in testosterone, luteinising hormone (LH) or the LH:Testosterone ratio or in sperm count or testicular apoptosis (Table 1).

### Effects of parental (F0) HFD on F1 offspring

At 19 weeks, adult males and females reared since weaning on CD or HFD were mated with opposite sex CD-fed controls to generate F1 offspring. No differences were observed in litter size, days taken to plug, gestation length, birthweight or proportion of males per litter in the F1 animals born to parents who had consumed a HFD or a CD (Supplementary Table 1). There were differences in the growth trajectories of the F1 offspring, with increased weight gain in F1 offspring of mothers that had consumed the HFD and in female offspring of HFD-fed males when compared to offspring of CD mothers (Fig. 1B). Bodyweight at sacrifice was increased for male (10%) and female (7%) F1 offspring born to HFD mothers (Table 2), although there were no differences in adiposity, or in leptin, insulin and glucose levels (Table 2). Testosterone was elevated by ~70% in the sons of HFD-exposed mothers although there was no difference in plasma LH or the LH:T ratio (Table 2).

### Grandparental diet affects F2 metabolic physiology in a parent-of-origin and sex-specific manner

To determine if grandparental exposure to HFD exerted effects on grandchildren (F2 offspring), representative F1 adult males and females derived from the relevant CD-exposed or HFD-exposed F0 parents were mated with opposite sex CD-fed controls to generate F2 offspring. No differences were observed in litter size, percentage of males per litter or birth-weights of F2 grand-offspring of CD or HFD fed rats (Supplementary Table 2). From 6 weeks of age, the F2 males whose maternal grandfather consumed a HFD were heavier than the comparable F2 male offspring of rats fed CD (Fig. 1C).

In adulthood, the adiposity index of the F2 male rats whose maternal grandfather consumed a HFD was 31% greater than control males (Table 3). There was an associated 97% increase in plasma leptin (Table 3) and evidence for decreased insulin sensitivity during the GTT, with insulin area under the curve increased by ~70% following a glucose challenge, although this was not statistically significant following Bonferroni adjustment (Table 3).

There was a trend for reduced plasma testosterone and increased LH levels in F2 males whose maternal grandfather had consumed a HFD (Table 3), which resulted in a significant three-fold increase in the LH:testosterone ratio in males of this group; this is indicative of compensated Leydig cell failure. There was no observable influence of grandpaternal diet on sperm count in F2 males (Table 3).

Female F2 animals derived from an HFD-fed grandparent showed no differences in body weight, adiposity or size of organs when compared to controls (Table 3).

### High fat diet does not alter the transcriptome of F0 male intra-testicular GCs

**Figure 2:**
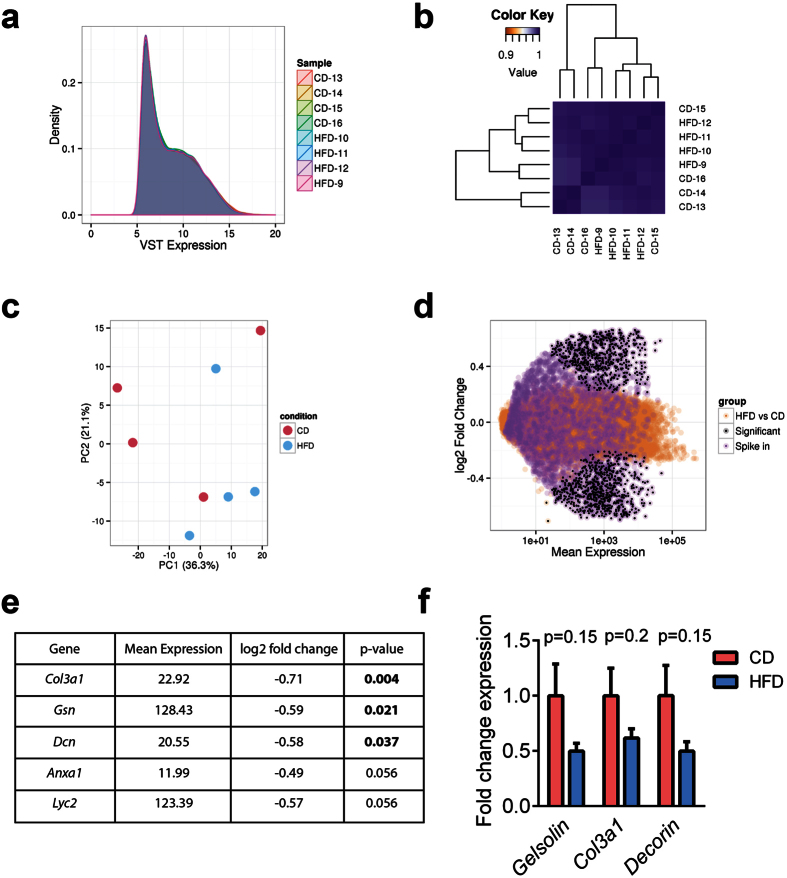
Effect of 14-week exposure to a high fat diet (HFD) or a control diet (CD) on the intra-testicular GC protein-coding transcriptome of F0 male rats. (**a**) The distribution of variance stabilising transformed (VST) expression of annotated protein-coding genes was unaffected by diet and was highly consistent across biological replicates. (**b**) Hierarchical clustering on the sample correlation matrix of gene expression indicated that the HFD and CD samples are highly similar. (**c**) Principal components analysis (PCA) was unable to distinguish samples according to diet. (**d**) Differential expression testing identified 3 protein-coding genes that were statistically significantly down-regulated in response to HFD (see **e**). A *post hoc* power analysis by simulation showed that the few HFD-induced changes were not due to a lack of statistical power given the same expression value and fold change range (Orange points = HFD vs. CD comparison; black points = statistically significantly differentially expressed genes between HFD and CD, including the simulated genes; purple points = simulated genes comparison). (**e**) The top 5 most differentially expressed genes in GCs of HFD-fed rats when compared with CD-fed rats. Mean expression indicates the expression level in GCs from CD animals, normalised for library size and averaged across 4 replicates; p-values were adjusted for multiple testing. (**f**) RT-qPCR validation analysis for the differentially expressed genes in (**e**). (Means ± SEM for n = 11–12).

**Figure 3:**
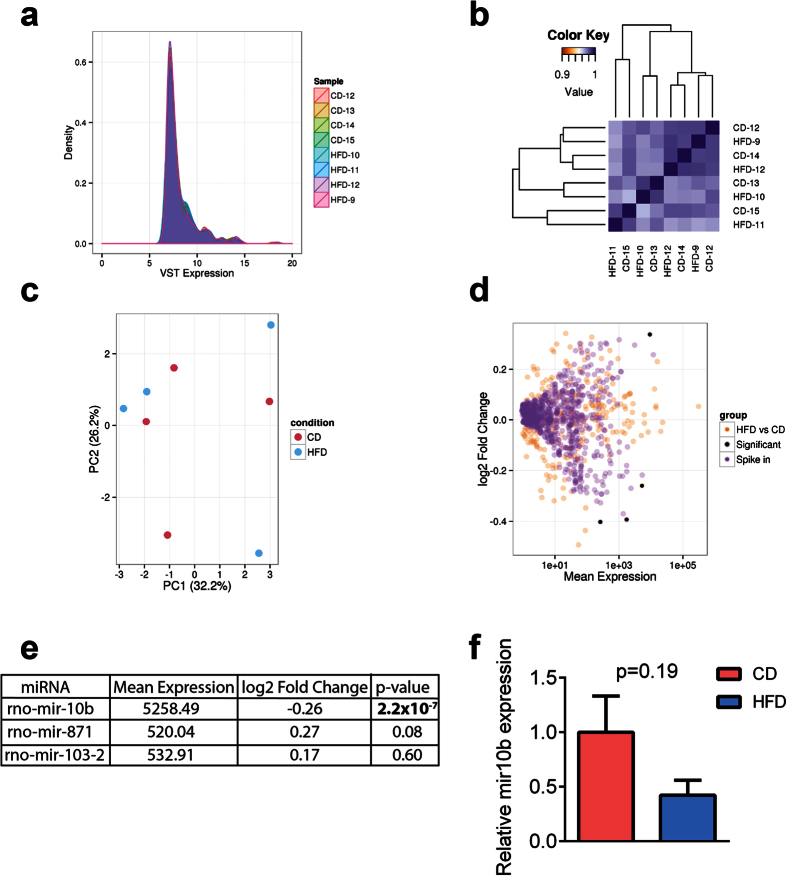
Effect of 14-week exposure to a high fat diet (HFD) or a control diet (CD) on regulatory miRNA expression in the intra-testicular GCs of male F0 rats. (**a**) The distribution of expression of annotated *Rattus norvegicus* miRNAs within GCs was unaffected by paternal diet. Expression was more variable between replicates than for the protein-coding mRNAs (Fig. 2), but showed that the majority of miRNAs were relatively lowly expressed (shown as Variance Stabilised expression). (**b**) Hierarchical clustering of miRNA expression showed that the HFD and CD samples had high similarity. (**c**) Principle components analysis (PCA) did not separate samples according to HFD or CD over the first two principal components, which together explained the majority of the variance in miRNA expression between samples. (**d**) Differential expression testing of miRNA expression identified a single miRNA (rno-mir-10b) that was significantly down-regulated in the HFD group compared with CD. This small but statistically significant change was mirrored in a *post hoc* power analysis by simulation that demonstrated a similar proportion of differentially expressed spike-in miRNAs (0.35% vs 0.48%) (Orange points = HFD vs. CD comparison; black points = statistically significantly differentially expressed genes between HFD and CD (including simulated genes); purple points = simulated gene comparison). (**e**) Top 3 most differentially expressed miRNAs in GCs of HFD-fed rats when compared with CD-fed rats. The miRNA rno-mir-10b is highly expressed and demonstrates a modest difference between HFD and CD groups. P-values were adjusted for multiple testing. (**f**) RT-qPCR validation analysis for the down-regulation of rno-mir-10b. Values are Means ± SEM for n = 8.

As F0 male exposure to HFD resulted in intergenerational effects in F2 male offspring, we investigated if HFD exposure altered the gene expression profile of intra-testicular GCs from F1 males. Purity of FACS sorted GCs was verified by qPCR for the GC specific protein *Vasa* and expression of *Sox9* (Sertoli cell-specific) and *3βHSD* (Leydig cell-specific) (Supplementary Figure 1). Total RNA was extracted from the GCs and underwent deep sequencing; 5.0–9.0 × 10^7^ reads per animal for RNA and 1.2–1.7 × 10^7^ reads for small RNA were uniquely aligned to the rat genome (rn5). The distribution of gene expression between rats on HFD and CD, and between biological replicates, was highly consistent for protein coding genes (Fig. 2A) and miRNAs (Fig. 3A) indicating little overall change in GC transcription in this model. Clustering and principal components analysis showed strong homogeneity between GCs isolated from HFD- and CD-exposed males (Figs 2B,C and 3B,C). Three protein coding mRNAs and 1 microRNA were down-regulated with statistical significance in GCs following HFD consumption; *collagen3a1*, *gelsolin* and *decorin* (Fig. 2E) and miRNA rno-mir-10b (Fig. 3E). Although each of these showed reduced expression in qPCR validation, these failed to reach statistical significance (Figs 2F and 3F).

There were no differentially expressed piRNAs or repeat elements when comparing GCs from CD- or HFD-exposed males (Supplementary Figure 2D). Both piRNA and repeat elements showed consistent distribution between treatment and biological replicates (Supplementary Figure 2A), and strong homogeneity was demonstrated by clustering analysis (Supplementary Figure 2B) and PCA (Supplementary Figure 2C).

Given recent evidence that dietary exposure can affect the abundance of specific tRNA fragments in mature spermatozoa^13^,^16^, the expression of tRNA derived species was examined. Our library preparation selected for small RNA species of 22–30 nt so we were only able to estimate expression (Sharma *et al*. found tRNA fragments to be 28–34 nt), however we were able to quantify 10 species of tRNA, none of which exhibited differential expression in the germ cells extracted from the testes of CD or HFD exposed animals. This is consistent with the findings of Sharma *et al*. who were unable to identify differential expression in testis tissue^13^.

### RNA-seq data from FACS sorted GCs was adequately powered to detect differences in protein coding gene expression

Given the few differentially expressed protein-coding genes and miRNAs in GCs of HFD-exposed F0 males, we next determined if our study design was adequately powered. We performed a *post hoc* power analysis in which data from the initial dataset were shuffled to generate a simulated dataset, selected for genes with differential expression of |log2fold| ≤ 2.0 (see Methods). This simulated dataset was analysed using the same approach as described for all other annotations. Our analysis revealed 2781 simulated genes with statistically significant differences in expression in this data set, suggesting the study was adequately powered to detect expression changes at a log2fold change ≤ 0.6 (Fig. 2D and Supplementary Figure 4). A similar *post hoc* power analysis was conducted on the miRNA data (Fig. 3D) which identified 3 differentially expressed miRNAs, a proportion of differentially expressed miRNAs similar to our original analysis (0.48% vs 0.35% respectively) (Supplementary Figure 4).

## Discussion

The aim of the present studies was to investigate if feeding rats a HFD results in metabolic or reproductive changes in subsequent generations and, if so, whether this might be mediated via altered gene expression in the GCs of the HFD-exposed parents (males in this case). Our data show that parental HFD exposure for a 16-week period can alter the metabolic phenotype of offspring and grand-offspring, with the most pronounced effects occurring when the maternal grandfather was exposed to a HFD. These effects occurred despite only modest changes in the adiposity of the HFD-exposed grandfathers. We did not detect any significant HFD-mediated changes in the transcriptome of the GCs from the testes of F0 male rats.

Consistent with previous studies, we found that exposure of the maternal grandfather to HFD had the greatest impact on bodyweight, adiposity and insulin resistance in grandsons^6^,^11^. The animal model data also agrees with epidemiological data from Överkalix, in Sweden, in which the environment of men during puberty predicted cardiovascular disease in grandsons, although in this instance via the paternal line^17^,^18^.

F2 males whose maternal grandfather had been exposed to HFD exhibited compensated Leydig cell failure, with an elevation in LH in the presence of normal testosterone levels. Although the relevance of compensated hypogonadism remains unclear, it has been associated with increased mortality^19^ and cardiovascular disease^20^,^21^. In humans, obesity is associated with hypogonadism^22^, although the causality of this is unclear^23^. The etiology of many cases of primary hypogonadism in men is unknown although there is some evidence that an altered *in utero* environment can program this effect in adulthood^24^.

Postnatal exposure of male rats to HFD resulted in metabolic changes in their grand-offspring, and previous studies have demonstrated changes in sperm RNA profiles following environmental exposures^6^,^14^ ; however, we found no differences in miRNA expression in intra-testicular GCs. This is in contrast to studies reporting alterations in miRNA in spermatozoa^6^,^25^, and suggests that HFD may affect the post-transcriptional stability of miRNA within maturing spermatozoa in the epididymis rather than in altered expression in the GC within the testis. Comparable studies in mice have shown that HFD exposure significantly altered the whole testis transcriptome^6^,^14^, with a proportion of transcripts showing altered expression in epididymal sperm. One key difference to the data presented here is that we excluded somatic cells from our analysis. Although this could suggest that such differences are due to the changes to somatic cells as a result of the nutritional exposure, Grandjean *et al*. went on to show that microinjection of miRNA mir-19b, which had increased expression in the testis and epididymal sperm of HFD fed mice, into one-cell embryos resulted in an altered metabolic phenotype mirroring that of offspring of HFD exposed fathers^14^. We found remarkable homogeneity in the RNA and small RNA-seq data from the intra-testicular germ cells of male rats exposed to CD or HFD, in a study which was powered to identify any changes in expression of log2fold change >0.6. This suggests that the changes identified may be species and/or experimental model specific. Unfortunately, due to difficulties in obtaining sperm populations with sufficient purity and abundance to interrogate miRNA profiles, we are unable to say if HFD-exposure induced changes in miRNA expression in mature epididymal sperm in our study. As several groups have now reported changes in expression of RNA species in epididymal spermatozoa following dietary interventions, and in the case of Sharma *et al*. in the absence of alterations in expression in testicular tissue^13^, we suggest that sperm maturation may be the stage of germ cell development most vulnerable to environmentally-induced perturbations.

Phenotypic changes may be transmissible across generations without changes in the GC transcriptome, for example in a model of maternal undernutrition, in which F2 mice exhibited an altered metabolic phenotype, the methylome of epididymal sperm was disrupted as a result of *in utero* undernourishment^12^ and in humans methylation of mature sperm DNA was altered in obese individiuals^26^. Such changes in the GCs in our model could have occurred without affecting transcription. Two further studies suggest that intergenerational effects of diet might be mediated by alterations in chromatin structure in mature spermatozoa^8^,^9^, which may occur in the absence of altered expression of mRNA in GCs, especially given that during spermiogenesis, the germline becomes largely transcriptionally inactive^27^.

The time-point(s) of importance for an environmental exposure to affect sperm, and the health of offspring and grand-offspring are unknown. Our results indicate that exposure to a HFD from weaning through puberty to adulthood is sufficient to program an effect in offspring, as has been shown in mouse models^11^,^28^. It is not clear how long such effects may persist; in mice, 7 weeks exposure to exercise or CD following 8 weeks exposure to HFD normalised insulin sensitivity in offspring, indicating some reversibility of the programmed phenotype^29^. One might speculate that a short-term, reversible effect would be more likely to arise from perturbations to sperm maturation than to spermatogonia, as has been suggested by studies that found programmed phenotypes following relatively short paternal exposures, for example as a consequence of exposure to 48 h high sugar in *Drosophila*^8^ and 24 h fasting in mice^30^. The timing of these acute exposures along with the results presented here, might point towards perturbations in epididymal sperm maturation, rather than effects on spermatogenesis. This is supported by evidence suggesting that tRNA/tRNA fragment accumulation in maturing spermatozoa may play a role in influencing offspring phenotype^13^,^16^. Thus our data add further weight to the argument that sperm maturation is most susceptible to environmental influences. Alternatively, our data might suggest a non-GC mediated transmission of the effects of HFD exposure, for example alterations in seminal fluid, which is important for establishing normal conception and healthy development^31^,^32^, and which is altered by obesity^31^,^33^.

Our study has a number of limitations. The HFD exposure in our study resulted in only modest weight gain, although it clearly implicates dietary fat, or the physiological response to it, as a factor resulting in changes in the health of offspring. The study utilised a semi inbred rodent line, which may result in greater variability between animals than studies in inbred mice. Furthermore, we cannot rule out genetic variation as a potential cause for the altered phenotype. However, intergenerational programmed effects have been reported in both inbred and outbred models^12^,^34^, and outbred strains are a better model of the human population. We have not further explored the epigenome in the purified GCs as, having found no changes to transcription, the relevance of any differences would be difficult to interpret; thus, we cannot discount possible epigenetic changes in regions in non-transcribed DNA in GCs. Finally, since we purified germ cells from the testis, we cannot account for any stage-specific GC effects, which could theoretically mask changes in gene expression; for example, if expression of a gene was increased in early spermatogonia but reduced in spermatids.

In conclusion, we show that postnatal exposure of male rats to HFD results in impaired metabolism in grandsons, a trait specifically transmitted down the maternal line. We did not detect significant changes in the intra-testicular GC transcriptome as a result of exposure to HFD that would explain the intergenerational effects. Further work is clearly necessary to discover mechanisms, to determine the time points at which males are most susceptible to HFD-induced changes, and establish if the effects are reversible. Given the rapid rise in the prevalence of obesity our data highlight that the environment of our immediate ancestors could play a role in this epidemic.

## Materials and Methods

### Study design and animal model

Studies were performed according to the Animals (Scientific Procedures) Act 1986 following specific approval from the UK Home Office (Project Licence 60/3962), following review by the University of Edinburgh Animal Research Ethics Committee. Animals were maintained under controlled lighting (lights on 0700-1900) and temperature (22 °C). Sprague Dawley GCS-eGFP rats^15^ (a gift from R. Hammer, University of Texas Southwestern Medical Center, USA) were mated in-house. Animals had access to water and diet *ad libitum* and were killed by CO_2_ inhalation followed by cervical dislocation.

30 male and 18 female 21-day-old founder (F0) rats were weaned onto either a high-fat, soya free diet (45% fat from lard) (HFD), or a matched control diet (10% fat) (CD) (Research Diets, NJ, USA) (for details see Supplementary Table 3) and weighed every 2 weeks. At 17 weeks, rats underwent metabolic testing. Two weeks later, 3 groups of virgin breeding pairs of F0 animals were established (n = 5–9), 1) mother CD/father CD, 2) father HFD/mother CD, and 3) mother HFD/father CD. Animals were used once for breeding. The days taken for a plug to be observed and the length of gestation were recorded. Following delivery, F1 litters were culled to eight pups (four males and four females where possible), and were weighed and weaned onto CD on day 21.

At 19 weeks, a female and male rat from each F1 litter (n = 4–7) were bred with a rat from the F1 control group to generate 4–5 F2 litters for each experimental arm and 7 F2 control litters. F2 litters were culled to 8 pups at birth and then culled to two animals per litter at 5–6 weeks of age. A total of 48 F2 males and 48 F2 females were thus derived (from 25 litters). Animals from the same treatment group were housed together with a maximum of 4 per cage and maintained throughout on CD.

### Metabolic testing

For F0 and F1 animals, one animal per litter underwent oral glucose tolerance testing (OGTT) (F0 n = 9, F1 n = 5–6). For F2 animals, two animals from each litter were tested (n = 8–14 from 4–7 litters). Following an overnight fast, at 09.00, blood was obtained by tail nicking into EDTA coated micro tubes (Starstedt, Germany) and plasma separated. 2 g/kg of 0.5 g/ml glucose solution (Sigma) was administered by oral gavage. Further blood was collected at 30 and 120 min.

For F0 animals, glucose was measured using a colorimetric kit (Cayman, USA) and for F1 and F2 with a kit (Alpha Laboratories Ltd., UK) adapted for use on a Cobas Fara centrifugal analyser (Roche, UK). Insulin was measured using a Rat Insulin ELISA kit (Mercodia, Sweden,). Fasting plasma triglyceride and cholesterol were determined using kits (Alpha Laboratories Ltd., UK and Olympus Diagnostics Ltd, UK, respectively), adapted for use on a Cobas Fara centrifugal analyser (Roche, UK). Terminal plasma leptin was measured using a Rat Leptin ELISA (Crystal-Chem, IL, USA).

### Testosterone and Luteinising Hormone (LH)

Plasma testosterone levels were measured at termination using an in-house radioimmunoassay described previously^35^. Plasma Luteinising Hormone (LH) was determined using an in house ELISA with the capture by a monoclonal anti-beta chain antibody (from Dr. Jan F Roser, University of California, USA) and a signal biotinylated anti-beta-chain monoclonal antibody (Medix, Finnland)^36^.

### Epididymal sperm count

Epididymides were dissected and the caput and cauda nicked once prior to placement into 5 ml F12:Ham’s (Life Technologies, UK) supplemented with 10% fetal calf serum (FCS) and 2% bovine serum albumin (BSA) (Sigma, UK). The epididymis was incubated for 1 hour at 37 °C with inversion at 30 mins. Sperm in the medium were counted using a modified Neubauer haemocytometer at ×40 magnification; 3–4 fields were counted per animal.

### Organ wet weights

Following sacrifice, pancreas, liver, testes, epididymal fat pads, and left retroperitoneal fat pads were dissected and weighed. Anogenital distance (AGD) was measured (using a 30 cm rule for F0 and F1 and digital callipers for F2) from the midpoint of the anus to the scroto-penile junction. For F2 animals, the penis was dissected and measured using callipers. Body length was measured using a 30 cm rule from the tip of the nose to the end of the rump.

### TUNEL staining

TUNEL staining used a modified protocol for the Promega DeadEnd kit (Promega). Testes were fixed in Bouin’s for 6 hours with bisection at 4 hours before paraffin embedding. 5 μm sections were dewaxed, rehydrated and washed in PBS, fixed for 15 min in 4% paraformaldehyde in PBS, treated with 20 μg/ml Proteinase K for 10 mins prior to further fixation in 4% paraformaldehyde. The remaining process was carried out as per manufacturer’s instructions. Slides were counterstained with DAPI prior to aqueous mounting. Four 40 × 10 × 10 tiled images were captured from each section using a Zeiss LSM 710 microscope. The number of positive pixels per tile was determined using ImageJ^37^.

### RNA-seq

Testes were decapsulated and minced in 5 ml ice cold Hanks Buffered Saline Solution with 0.1% Collagenase IV (Sigma, UK). The suspension was dissociated before incubation at 37 °C with gentle rotation for 10 min, and passage through a 40 μm cell strainer. Cells were washed 3x by centrifugation at 500 *g* for 5 min and resuspended in 10 ml 2% FCS (Invitrogen, UK). Samples were kept on ice until FACS on a BD Aria II, gating for expression of eGFP. 6 × 10^6^ sorted cells were centrifuged and RNA immediately extracted using the Qiagen miRNAeasy mini kit (Qiagen, UK) according to manufacturer’s instructions. Quality and purity of total RNA was verified using spectrophotometry and the Agilent RNA 6000 nano kit before library preparation (Illumina RNA library prep kit v2 and the Illumina Truseq small RNA kits (Illumina, CA, USA)). The small RNA library preparation selected for RNA species with length 22–30 nt. Sequencing small RNA (smRNA) and RNA was performed on the Illumina HiSeq2500 (Edinburgh Genomics, Edinburgh, UK). Intended library size for total RNA was 37.5 million single end reads of 125 bp and for small RNA was 20 million single end reads of 50 bp. Quality of the sequencing was verified using FastQC. Adaptor contamination was removed from the smRNA-seq libraries using trimmomatic to a minimum length of 25 nucleotides^38^. SmRNA-seq reads were aligned to the *Rattus norvegicus* genome version rn5 using Butter with default parameters^39^. Differential expression was determined using DESeq2^40^. RNA-seq libraries were aligned to Rn5 using Star v2.3.0^41^ on a custom-built splice junctions database based on Rn5 ensembl73 protein-coding annotations, with the maximum proportion of mismatches over the read length of 0.05 and minimum mapping read length of 125 nts. As positive controls, liver and testis RNA-seq data from the Rat bodymap were subject to the same bioinformatic pipeline^42^ (Supplementary Figures 3A,D). All raw RNA-seq data, and processed count tables are archived at the Gene Expression Omnibus with accession number GSE80721. The code used to perform read quality control (pipeline_readqc.py) and short read alignment (pipeline_mapping.py) can be found at https://github.com/CGATOxford/CGATPipelines.

### Sample clustering and principal components analysis

Variance stabilising transformed (VST) read counts for protein-coding genes, miRNAs, repeats and piRNAs were calculated using DESeq2. Between-sample Pearson correlations (r) were calculated using VST counts and used to hierarchically cluster samples using average linkage clustering, with distances defined as 1 − | r |. Principal components analysis (PCA) was performed on scaled and centred VST counts using the R *prcomp* function. Sample clustering was visualised using the R gplots package function *heatmap.2*, and PCA results were plotted using the R grammar of graphics package, *ggplot2*.

### Differential expression testing

Uniquely aligned reads were counted over rn5 genomic annotations (ensemble v73 protein-coding genes, miRBase miRNAs^43^, piRNAQuest piRNAs^44^ and repBase repeat classes^45^) using featureCounts^46^. Genomic annotations with a mean read count <1 across all samples were excluded from analysis. This resulted in the differential expression testing of 18,025 protein-coding genes, 285 miRNAs, 7390 piRNA annotations and 522 repeat classes. Statistical testing was carried out separately for protein-coding genes, miRNAs, piRNAs and repeat elements. Differential expression testing was performed using a negative binomial general linear model, regressing genomic annotation read counts on diet, adjusted for library size, in the Bioconductor package DESeq2^40^. P-values were calculated based on the Bayesian shrinkage moderated log2 fold changes by a Wald test with H_0_: log2 fold change = 0, H_A_: log2 fold change ≠ 0 and adjusted for multiple testing using the Benjamini & Hochberg procedure^47^.

### Power analysis

Evaluation of statistical power was determined by simulation. For any given annotation, read counts were randomly and iteratively shuffled to generate a simulated dataset of read counts, preserving the experimental design, i.e. counts were shuffled between different genes, but not different treatment groups. Shuffled annotations were retained with |log2 fold change| ranges 0.0–2.0 for protein-coding genes, and 0.0–0.5 for miRNA annotations. These ranges were selected to reflect the observed fold changes in the experimental data. The counts tables derived from the shuffled data sets were used as input into the same differential expression testing procedure described above. The shuffled counts tables were generated using the *counts2counts.py* Python script in the CGAT code collection^48^, found at https://github.com/CGATOxford/cgat. To generate Figs 2D and 3D, the relevant table of simulated counts were spiked into the table of relevant annotation counts, and the differential expression analysis was performed on the combined table. Statistical power was calculated in bins of 0.1 as the proportion of statistically significantly differentially expressed annotations, relative to all tested annotations of that class (i.e. either miRNAs or protein-coding genes). The R code to generate the power curves and all other code to perform differential expression testing can be found at https://github.com/MikeDMorgan/proj035, including the counts tables on which the power analysis in Supplementary Figure 4 was performed.

### qPCR validation of sequencing

cDNA was prepared using SuperScript VILO (Invitrogen) as per the manufacturer’s instructions. The most stably expressed genes from the RNA-seq were used to determine reference genes using normfinder^49^. Expression was thus calculated relative to the mean expression of *Ldha* and *Ropn1* *L*. qPCR was performed on the ABI Prism Sequence Detection System (Applied Biosystems). Expression was determined using the primers and universal probes (Roche, UK) in Supplementary Table 4. For miRNA, the ABI TaqMan miRNA assay for rno-mir-10b was used according to manufacturer’s instructions; snoRNA-U6 was used as control. RNA from two independent cohorts of rats was used. All samples were analysed in triplicate.

Validation of the purity of sorted GCs was achieved by qPCR as above but with expression compared to RNA from adult rat testis (Ambion), using 18S as internal control.

### Statistics

For experiments examining F0 founders, outcomes were analysed using linear mixed model with diet and sex as fixed factors and cohort as a random factor. For analysis of F1 and F2 data, a mixed linear model was used with group and sex as fixed factors and litter number as a random factor. Data are presented either as a % difference to the control group or, as mean ± SEM with total animals as the denominator. For bodyweight, a mixed linear model was used with group and sex as fixed factors, litter as a random factor and weight as a repeated measure with an autoregressive covariance structure. Goodness of fit of these models was checked using the maximum likelihood method and comparison of −2 log likelihood information criteria for the lowest value. *Post hoc* Bonferroni analysis was conducted to account for multiple comparisons. Where no differences were determined by Bonferroni adjustment, but an interaction was identified, least significant difference analysis was conducted as indicated in the results tables. Levels of significance were set at alpha = 0.05, statistics were computed using SSPS version 19 (IBM, USA).

## Additional Information

**How to cite this article**: Chambers, T. J. G. *et al*. High-fat diet disrupts metabolism in two generations of rats in a parent-of-origin specific manner. *Sci. Rep.* **6**, 31857; doi: 10.1038/srep31857 (2016).

## Supplementary Material

Supplementary Information

## Figures and Tables

**Table 1:** Effect of feeding with a high fat (HFD) or control diet (CD) for 14 weeks on the metabolic and reproductive phenotype of F0 males.

N (litters)	Control diet	High fat diet	p
	7–15(6)	7–15(6)	
	Mean ± SEM	Mean ± SEM	
Body size			
Weight (g)	416 ± 14	**454** ± **14**	**0.003**
Body length (cm)	23.95 ± 0.25	24.10 ± 0.31	0.565
Organ weights			
Pancreas weight/bodyweight (mg/g)	1.9 ± 0.09	1.8 ± 0.08	0.416
Liver/bodyweight (mg/g)	38.3 ± 1.2	**31.0** ± **0.9**	**<0.001**
Adipose weights			
Retroperitoneal Fat/bodyweight (mg/g)	4.7 ± 0.03	**6.9** ± **0.04**	**<0.001**
Gonadal fat/bodyweight (mg/g)	6.3 ± 0.3	**8.3** ± **0.3**	**<0.001**
Adiposity Index (mg/g)	11.0 ± 0.7	**15.2** ± **0.7**	**<0.001**
Biochemistry			
Leptin (ng/ml)	1.67 ± 1.28	5.25 ± 1.21	0.054
Insulin AUC μg/l.min	96.4 ± 11.1	**144.7** ± **10.1**	**0.003**
Glucose AUC mM.min	647.1 ± 22.7	672.2 ± 17.6	0.4
Triglycerides mM	0.81 ± 0.06	0.73 ± 0.07	0.719
Cholesterol mM	1.53 ± 0.07	1.58 ± 0.10	0.414
Reproduction			
AGD (mm)	46.91 ± 1.29	45.93 ± 0.80	0.52
Testis weight (g)	1.79 ± 0.06	1.73 ± 0.05	0.334
Penis length (mm)	10.69 ± 1.22	12.14 ± 0.11	0.25
Sperm count (10^6^)	19.10 ± 2.60	17.50 ± 1.44	0.598
Testosterone (ng/ml)	5.14 ± 1.53	7.31 ± 0.66	0.075
LH (ng/ml)	0.37 ± 0.05	0.73 ± 0.3	0.322
LH:T	0.08 ± 0.01	0.09 ± 0.03	0.236
% motile sperm	32.36 ± 4.37	26.64 ± 2.46	0.277
TUNEL (pixels)	1111 ± 414	1451 ± 263	0.504

Data are derived from post-mortem dissection at 19 weeks of age. Biochemical data derives from 09.00 fasting plasma obtained during glucose tolerance testing at 17 weeks of age. Data were analysed by linear mixed model with diet as fixed factor and cohort as a random factor with post hoc Bonferroni analysis. Values significantly different (p < 0.05) from control (CD) are shown in bold.

**Table 2:** Phenotypic analysis of F1 offspring of F0 mothers and fathers that were fed a high fat (HFD) or control diet for 14 weeks.

Sex	Male			Female			
Group	Control	Maternal HFD	Paternal HFD	Control	Maternal HFD	Paternal HFD	p
N (litters) unless stated below	19–32 (9)	13–19 (5)	10–17 (5)	10–17 (9)	14–20 (5)	13–21 (5)	
	Mean ± SEM	Mean ± SEM	Mean ± SEM	Mean ± SEM	Mean ± SEM	Mean ± SEM	
Body size							
** **Weight (g)	434 ± 4	**478** ± **10**	431 ± 8	260 ± 5	278 ± 4	270 ± 4	**0.003**
** **Length (cm)	23.8 ± 0.2	24.1 ± 0.3	23.5 ± 0.3	21.2 ± 0.2	21.1 ± 0.2	21.2 ± 0.2	0.114
** **AGD (mm)	47.8 ± 0.5	45.9 ± 2.2	44.8 ± 0.8	21.3 ± 0.3	19.3 ± 0.8	18.58 ± 0.8	0.945
Adiposity							
** **Gonadal fat/bw (mg/g)	7.7 ± 0.3	7.6 ± 0.3	6.7 ± 0.4	6.3 ± 0.3	7.2 ± 0.7	6.2 ± 0.5	0.062
** **Retroperitoneal fat/bw (mg/g)	5.3 ± 0.3	5.0 ± 0.4	4.5 ± 0.4	5.1 ± 0.3	4.9 ± 0.6	3.7 ± 0.3	0.177
** **Adiposity index (mg/g)	12.9 ± 0.6	12.6 ± 0.7	11.2 ± 0.7	11.4 ± 0.5	12.1 ± 1.2	10.0 ± 0.6	0.062
Biochemistry							
** **Leptin (ng/ml)(n = 5)	1.30 ± 0.29	1.72 ± 0.53	0.94 ± 0.12				0.346^
** **Insulin AUC (μg/l.min) (n = 5)	108.6 ± 21.6	76.65 ± 11.1	77.9 ± 14.7	51.39 ± 10.4	65.7 ± 13.3	47.05 ± 9.8	0.249
** **Glucose AUC (mM.min) (n = 5)	984 ± 32.4	931.2 ± 36.5	951 ± 39.3	931.2 ± 36.5	938.4 ± 24.0	904 ± 73.3	0.846
Reproduction							
** **Penis length (mm)	12.1 ± 0.08	11.8 ± 0.18*	11.7 ± 0.16*				**0.023^**
** **Gonad weight (g)	1.90 ± 0.02	2.00 ± 0.07	1.90 ± 0.07	0.08 ± 0.00	0.08 ± 0.00	0.12 ± 0.02	0.148
** **Testosterone (ng/ml) (n = 5)	4.22 ± 0.64	**7.38** ± **0.90**	2.73 ± 0.51				**0.002^**
** **LH (ng/ml) (n = 5)	0.38 ± 0.06	0.34 ± 0.04	0.26 ± 0.04				0.255^
** **LH:T ratio (n = 5)	0.10 ± 0.03	0.05 ± 0.01	0.11 ± 0.03				0.234^

Data are derived from post-mortem dissection at 19 weeks of age. Biochemical data derives from 09.00 fasting plasma obtained during glucose tolerance testing at 17 weeks of age. Data was analysed by linear mixed model with group and sex as fixed factors and litter as a random factor with post hoc Bonferroni analysis. Values significantly different (p < 0.05) from control are shown in bold.

^*^Indicates different only with least significant difference analysis (not taking multiple testing into account). ^Indicates sex not used as a fixed factor as data only available for males.

**Table 3:** Phenotypic analysis of F2 offspring of grandparents (F0 mothers and fathers) that were fed a high fat (HFD) or control diet for 14 weeks.

	Male					Female					
	Control	Maternal grandfather	Maternal grandmother	Paternal grandfather	Paternal grandmother	Control	Maternal grandfather	Maternal grandmother	Paternal grandfather	Paternal grandmother	p
N (litters)	14 (7)	8 (4)	8 (4)	10 (5)	10 (5)	14 (7)	8 (4)	8 (4)	10 (5)	10 (5)	
Body size											
** **Weight (g)	416 ± 5.2	448 ± 13.4	446 ± 8.8	412 ± 11.4	433 ± 9.5	268 ± 7.6	277 ± 5.4	269 ± 7.5	262 ± 5.9	271 ± 5.3	0.124
** **Length (cm)	23.3 ± 0.1	23.5 ± 0.2	23.5 ± 0.2	22.8 ± 0.1	23.4 ± 0.2	21.1 ± 0.2	20.9±0.1	21.0 ± 0.2	20.7 ± 0.2	21.1 ± 0.2	0.613
Organ weights											
** **Liver/bw (mg/g)	43.2 ± 0.8	40.8 ± 0.5	42.3 ± 1.4	42.8 ± 1.2	44.4 ± 1.4	38.9 ± ± 0.9	37.0 ± 1.5	34.3 ± 1.5	38.4 ± 1.4	37.6 ± 1.1	0.24
** **Pancreas/bw (mg/g)	2.6 ± 0.1	2.4 ± 0.2	2.1 ± 0.1	2.3 ± 0.1	2.2 ± 0.1	3.5 ± 0.1	3.5 ± 0.2	3.8 ± 0.4	4.0 ± 0.2	3.0 ± 0.2	**0.007**
Adiposity											
** **Retroperitoneal fat/bw (mg/g)	4.8 ± 0.4	**6.6** ± **0.5**	5.1 ± .4	6.3 ± ± 0.5	6.1 ± 0.4	4.1 ± 0.5	3.6 ± 0.2	3.9 ± 0.7	4.9 ± 0.3	5.2 ± 0.3	**0.003**
Gonad fat/bw(mg/g)	6.6 ± 0.3	8.4 ± 0.6*	7.0 ± 0.2	7.9 ± 0.5	6.9 ± 0.4	5.9 ± 0.5	5.2 ± 0.3	5.9 ± ± 0.4	6.3 ± 0.4	6.3 ± 0.5	**0.017**
** **Adiposity index (mg/g)	18.0 ± 1.0	**23.4** ± **1.7**	19.0 ± 0.7	22.0 ± 1.3	19.9 ± 1.0	16.0 ± 1.3	14.0 ± 0.6	15.7 ± 1.3	17.4 ± 0.9	17.8 ± 1.2	**0.012**
Biochemistry											
** **Insulin AUC (μg/l.min)	90.2 ± 10.7	148.7 ± 18.2*	104.0 ± 7.1	119.0 ± 13.3	128.5 ± 18.6	60.3 ± 7.4	86.2 ± 16.2	48.4 ± 2.9	103.3 ± 8.4	72.0 ± 11.9	**0.016**
** **Glucose AUC (mM.min)	964 ± 18.5	904 ± 6.6	947 ± 36.5	954 ± 10.7	966 ± 6.4	947 ± 24.1	931 ± 18.9	921 ± 27.7	1146 ± 13.4	978 ± 9.1	0.15
** **Leptin (ng/ml)	2.5 ± 0.3	**5.0** ± **0.8**	3.3 ± 0.2	3.6 ± 0.5	2.9 ± 0.4	1.8 ± 0.2	1.7 ± 0.2	1.9 ± 0.2	1.6 ± 0.1	1.9 ± 0.2	**0.001**
** **Cholesterol (mM)	1.08 ± 0.03	1.16 ± 0.05	1.03 ± 0.07	1.18 ± 0.05	1.03 ± 0.04	1.02 ± 0.04	1.10 ± 0.08	1.06 ± 0.06	1.25 ± 0.06	1.08 ± 0.07	0.474
** **Triglycerides (mM)	1.08 ± 0.05	1.3 ± 0.189	1.54 ± ± 0.13	1.16 ± 0.10	1.27 ± 0.12	0.82 ± 0.05	0.78 ± 0.04	1.21 ± 0.12	0.83 ± 0.07	1.14 ± 0.13	0.106
Reproduction											
** **LH (ng/ml)	0.6 ± 0.1	1.2 ± 0.2	0.7 ± 0.7	1.0 ± 0.2	1.0 ± 0.2						0.250^
** **Testosterone(ng/ml)	8.5 ± 0.5	6.1 ± 0.8	8.6 ± 1.4	8.7 ± 1.4	7.80 ± 0.6						0.270^
** **LH:T ratio	0.07 ± 0.01	**0.21** ± **0.04**	0.09 ± 0.02	0.13 ± 0.04	0.12 ± 0.02						**0.017^**
** **Penis Length (mm)	12.1 ± 0.1	12.3 ± 0.2	12.5 ± 0.2	12.3 ± 0.2	12.3 ± 0.1						0.614^
** **Sperm count (10^6^)	6.0±12	36.3 ± 7.5	22.4 ± 7.3	33.2 ± 8.8	23.3 ± ± 7.7						0.629^
** **AGD (mm)	48.6 ± 0.7	49.5 ± 0.9	49.9 ± 1.1	47.8 ± 0.8	50.9 ± 0.6	21.3 ± 0.5	23.1±0.2	21.7 ± ± 0.4	22.4 ± 0.4	22.7 ± 0.4	0.121

Data are derived from post-mortem dissection at 19 weeks of age. Biochemical data derives from 09.00 fasting plasma obtained during glucose tolerance testing at 17 weeks of age. Data was analysed by linear mixed model with group and sex as fixed factors and litter as a random factor with post hoc Bonferroni analysis. Comparing groups within each sex, values significantly different (p < 0.05) from control are shown in bold.

^*^Indicates different only with least significant difference analysis (not taking multiple testing into account). ^Indicates sex not used as a fixed factor as data only available for males.
