# Supplementary material for: High-fat diet disrupts metabolism in two generations of rats in a parent-of-origin specific manner
Source: Sci Rep. 2016 Aug 23;6:31857. doi: 10.1038/srep31857 (PMC4994008; doi:10.1038/srep31857)
Supplement: Supplementary Information [file srep31857-s1.pdf]

High-fat diet disrupts metabolism in two generations of rats in a parent-of-origin specific manner without affecting the intra-testicular germ-cell transcriptome

Chambers TJG, Morgan MD, Heger AH, Sharpe RM, Drake AJ.

Supplementary information

Figure S1 Validation of the purity of FACS-sorted GCs.
Relative expression (RT-qPCR) of the cell specific genes Vasa (GCs), Cdkn1b and Sox9 (Sertoli cells) and 3βHSD (Leydig cells) compared to whole testis are shown (Means ± SEM, N=4).

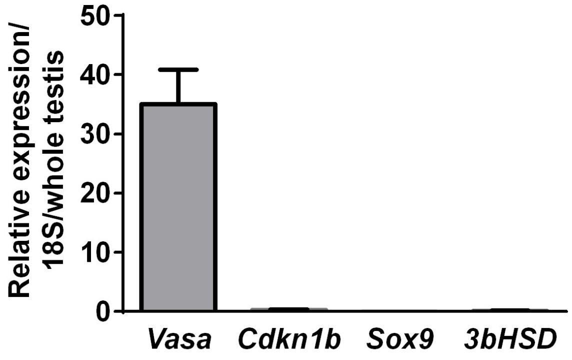

Figure S2 Effect of 14-week exposure to a high fat diet (HFD) or a control diet (CD) on the GC retrotransposon, repeat element and piRNA transcriptome of F0 male rats

A. The distribution of variance stabilising transformed (VST) expression values for annotated *Rattus norvegicus* piRNAs and expressed repeat elements was unaffected by diet and was highly consistent across biological replicates. The bimodal distribution of repeats expression was more similar to that of protein-coding genes than either miRNAs (Figure 3) or piRNAs. **B**. Hierarchical clustering of expression correlation of piRNAs (top) and repeat elements (bottom) between samples indicated that the HFD and CD samples were highly similar. **C**. Principal component analysis (PCA) across expressed piRNAs (top) and repeats (bottom) was unable to distinguish between the transcriptomes. **D**. Differential expression testing detected no differences in either the piRNA (top) or repeat element (bottom) expression.

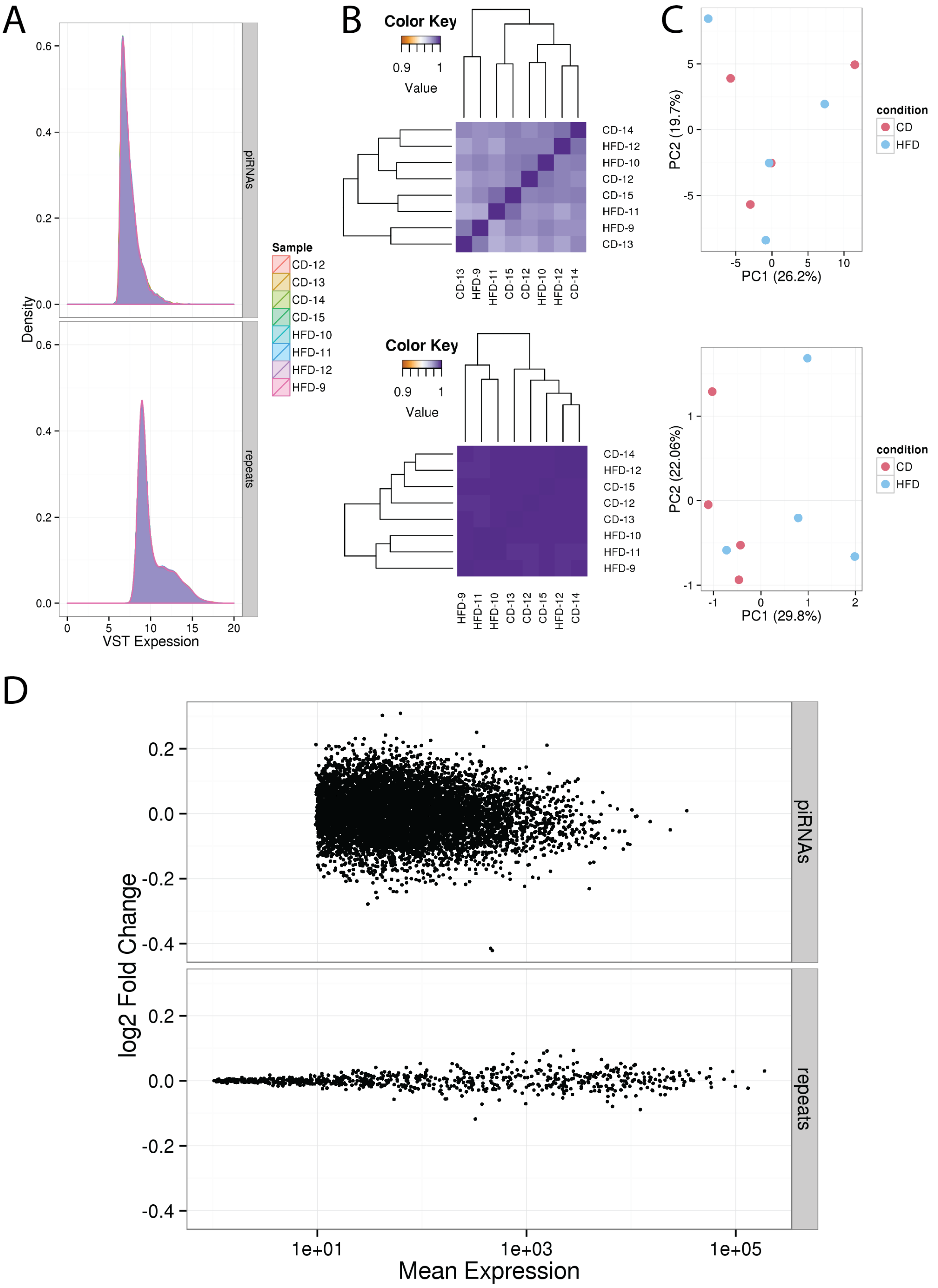
A
B
C
D
Color Key
Value
Sample
condition
CD
HFD
piRNAs
repeats
Density
VST Expression
PC1 (26.2%)
PC2 (19.7%)
PC1 (29.8%)
PC2 (22.06%)
log2 Fold Change
Mean Expression

Figure S3 Rat bodymap data demonstrate large tissue differences in mRNA expression between liver and testes

A. Distribution of expression values for annotated *Rattus norvegicus* protein-coding mRNAs displayed obvious differences between tissues. **B**. Hierarchical clustering of protein-coding gene expression separated samples by tissue type, in contrast to that found for comparison of CD- and HFD-fed rats (Figures 4B, 5B and supplementary figure 2B-3). **C**. Principal component analysis across expressed protein-coding genes differentiated between liver and testis samples, with 90% of the variation in expression explained by tissue differences. **D**. Differential expression testing detected large differences in gene expression between liver and testis tissue in the Rat Bodymap dataset that was not observed in the transcriptome components of the GCs of rats fed CD or HFD diets (red dots indicate significance with adjusted p-value ≤ 0.05).

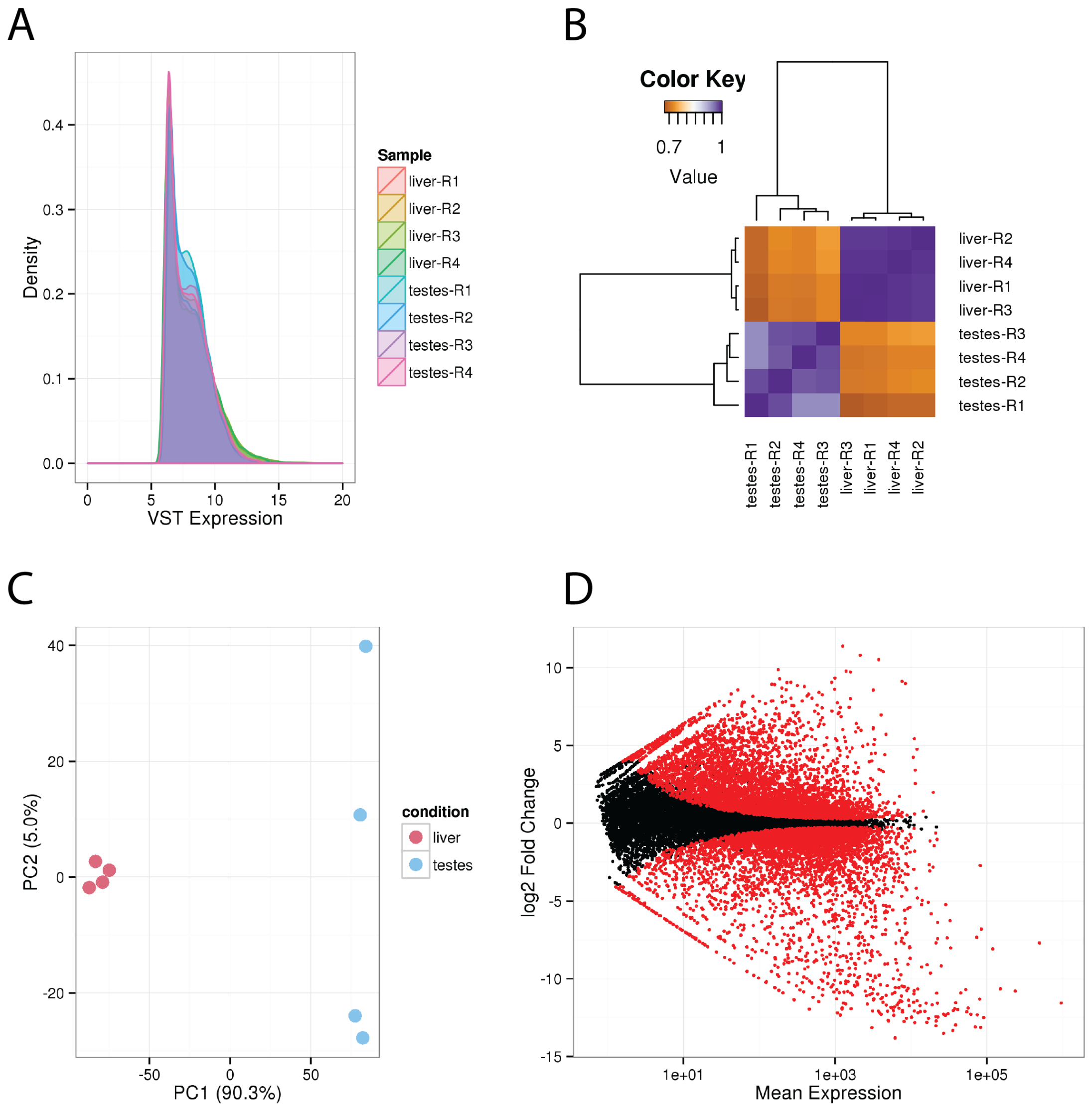

Figure S4 Comparison of RNAseq data from FACS sorted GCs in the present study with the top 50 up- and down-regulated genes identified from microarray analysis of whole testis extracts from mice following exposure to HFD from Fullston *et al.* (ref. 6). Spearman R correlation was used to compare the two data sets.

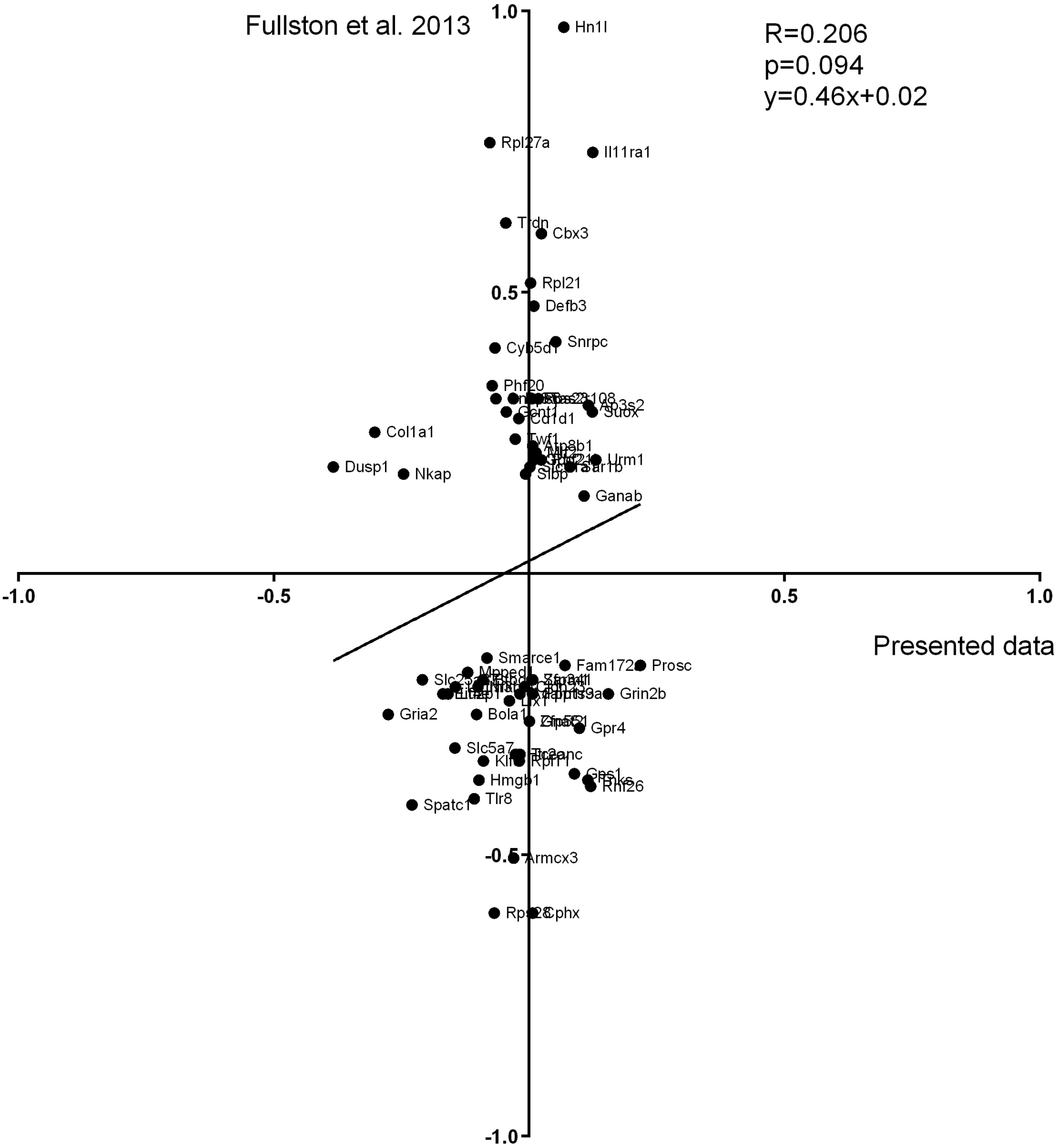

Figure S5 Power curves of spike-in experiments. **A**. Spike-in analysis of protein-coding genes demonstrated sufficient statistical power (80%) to detect estimated absolute log2 fold changes greater than ~|0.4| (horizontal grey line). The smallest observed estimate was ~|0.6|, shown by the vertical red line, indicating there was sufficient statistical power to detect a wide range of log fold changes. **B**. Spike-in analysis of miRNA data showed lower statistical power for the smallest observed difference, but there was sufficient power (grey line) at a similar level as for the protein-coding genes (~|0.4|). This suggests the sample size used in this study would have sufficient power to detect any observations above this threshold.

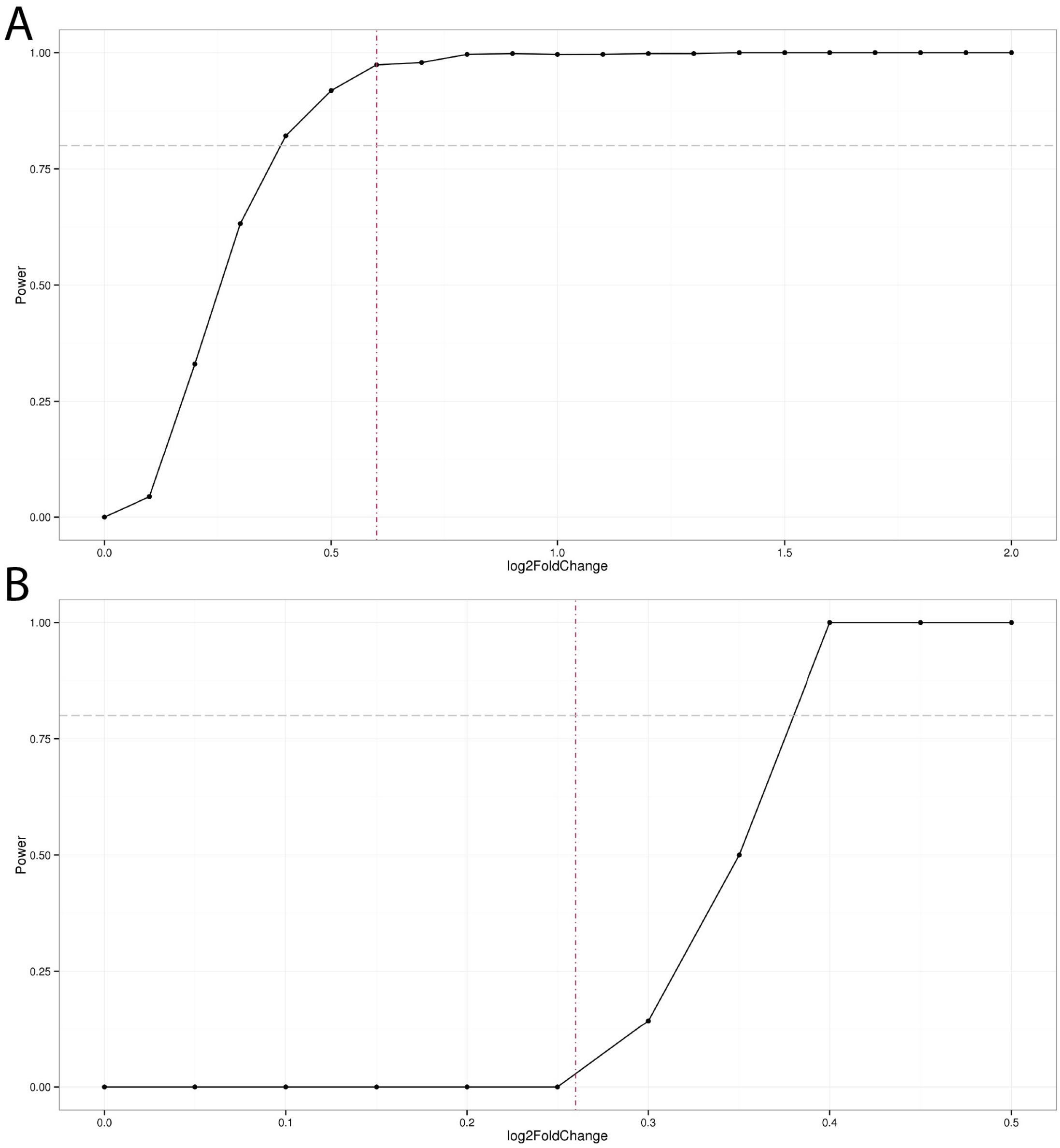

	HFD	CD
Research Diets ™ #	D06071701	D06072701
Cysteine (% w/w)	0.35	0.28
Casein (% w/w)	23.31	18.96
Corn-starch (% w/w)	8.48	29.86
Maltodextrin (% w/w)	11.65	3.32
Sucrose (% w/w)	20.14	33.17
Cellulose (% w/w)	5.83	4.74
Corn oil (% w/w)	2.91	2.37
Mineral mix (% w/w)	1.17	0.95
Vitamin mix (% w/w)	1.17	0.95
Lard (% w/w)	20.68	1.9
calories/100g	473	385
% of total energy from carbohydrate	35	70
% of total energy from protein	20	20
% of total energy from lipid	45	10

Table S1. Composition of control (CD) and high fat (HFD) - soya free diets, which were obtained from Research Diets; some of the carbohydrate was replaced by fat (lard) in the HFD.

	CD	Mother HFD	Father HFD	
litters	5	5	5	
	Mean±SEM	Mean±SEM	Mean±SEM	p
Litter size	10.20±1.39	12.60±0.87	12.40±0.75	0.236
Birthweight (g)	6.85±0.14	6.67±0.22	6.64±0.32	0.794
% males per litter	48.2±10.7	29.4±3.4	48.5±3.5	0.114
Days to plug	5.33±1.15	3.17±0.17	3.50±0.67	0.136
Gestation (days)	22.8±0.2	22.5±0.2	22.6±0.2	0.638

Table S2. Demographics of F1 litters born to F0 rats in which the mother or father had been exposed for 14 weeks to a control (CD) or high fat diet (HFD).

	Control	Maternal Grandfather	Maternal Grandmother	Paternal Grandfather	Paternal Grandmother	
Litters	7	4	4	5	5	
	Mean±SEM	Mean±SEM	Mean±SEM	Mean±SEM	Mean±SEM	p
Birthweight (g)	6.93±0.24	6.86±0.42	6.89±0.31	7.05±0.32	6.85±0.19	0.986
% male pups	46.5±2.8	58.2±4.0	46.0±5.6	55.0±8.4	58.6±7.6	0.384
Litter size	12.57±0.48	12.5±1.66	12.25±1.49	11.8±0.58	11.2±2.13	0.934

Table S3. Demographics of F2 litters according to the diet of the maternal or paternal grandparent. Top row indicates which grandparent consumed a high fat diet (HFD). Control indicates grandparents and parents consumed the CD. Animals consumed a CD unless otherwise stipulated.

**Primer**	**F**	**R**	**Probe**
Col3a1	cctgcaggaaaggatgga	gaggtccaggcagtccac	80
Dcn	ctccgagtggtgcagtgtt	gcaatgttgtgtcaggtgga	115
Gsn	ctggccaagctctacaaggt	agccacgagggagactgac	16
Ldha	gatctcgcgcacgctact	cacaatcagctggtccttgag	129
Ropn1L	catcctcaagcagttcacca	tacgggaagtgggtctcct	121
vasa	cattcagaagaggtgggagaga	tgctggtttcctagaaccaaa	77
3bHSD	gaccagaaaccaaggaggaa	ctggcacgctctcctcag	105
Sox9	atcttcaaggcgctgcaa	cggtggaccctcagattg	63

Table S4. Primers and probes used for qPCR
